# Probabilistic reliability assessment of reservoir-area colluvial landslides considering rotated spatial variability of geotechnical parameters

**DOI:** 10.1371/journal.pone.0340400

**Published:** 2026-02-04

**Authors:** Haifu Tang, Zhile Xu, Quan Zhao, Licheng Wu, Long Qin

**Affiliations:** 1 College of Civil Engineering, Guizhou University, Guiyang, China; 2 Juli Sling Co., Ltd., Baoding, Hebei, China; 3 Guizhou Geological Engineering Survey and Design Research Institute, Guiyang, China; China Construction Fourth Engineering Division Corp. Ltd, CHINA

## Abstract

Slope reliability analysis often assumes isotropic or anisotropic random fields with horizontal orientation to characterize the spatial variability of soil parameters. However, this neglects the influence of rotated anisotropic spatial variability, leading to conservative and unrealistic failure probability estimates. To overcome this limitation, we propose a novel method based on the Hierarchical Recurrent Highway Network (HRHN) with attention mechanisms. This method is applied to a time-dependent reliability assessment of the Baishuihe landslide. By incorporating the spatial variability of geotechnical properties—especially the direction of maximum fluctuation—the study constructs both the most adverse and favorable extreme scenarios, enabling the exploration of failure probability evolution under reservoir drawdown and rainfall infiltration. Compared with traditional horizontally anisotropic random fields, the proposed model produces a range of failure probabilities rather than a single curve. This interval range—formed by maximum and minimum failure probabilities—better captures the uncertainty of the model and accounts for external factors such as rainfall and geological changes. Our approach offers a more comprehensive and realistic perspective for geotechnical risk assessment.

## 1. Introduction

Landslides are among the most common geological hazards in China and pose a significant threat to human life and property. Preventing landslides has long been a primary focus in the fields of geotechnical engineering and engineering geology [1,2,3,4]. Traditional landslide reliability analyses typically assume that system parameters are time-invariant random variables. However, with the advancement of research, an increasing number of scholars have recognized that slope stability is inherently a time-evolving stochastic process, meaning landslide reliability exhibits time-dependent characteristics [5]. In recent years, studies on time-dependent reliability of landslides have developed rapidly in various aspects, including uncertainty quantification [6,7,8,9] and advanced surrogate modeling techniques [10,11,12].

It is well known that rainfall infiltration and reservoir water level fluctuations are among the most significant environmental factors affecting landslides in reservoir areas [13,14,12]. Studies have shown that the periodic fluctuation of reservoir water levels can significantly alter the hydrogeological conditions of a slope, thereby affecting its stability. Specifically, during periods of rapid drawdown, the failure probability of a slope tends to be overestimated, whereas under slower drawdown conditions, the reliability changes more gradually and smoothly [5]. For example, [15] proposed a time-dependent reliability analysis method for unsaturated slopes subjected to rapid reservoir drawdown, based on a typical reservoir slope model. By constructing numerical simulation scenarios and introducing intelligent surrogate models to improve the efficiency of Monte Carlo simulations, the study investigated the time evolution of failure probability and the sensitivity of key influencing parameters. Similarly, [12] developed a reliability assessment approach for weak-interlayered reservoir slopes, incorporating factors such as water level fluctuation, wetting-drying cycles, and seismic loading, based on random field theory. This method was used to quantify long-term failure probabilities of slopes during service life. Due to ease of implementation, most of the above studies treat rock and soil parameters as random variables. Consequently, few studies have incorporated the spatial variability of geotechnical parameters. In this regard, random field models offer an effective framework for reliability analysis by accounting for the spatial heterogeneity of soil and rock properties [16,17].

If the spatial variability of rock and soil is not considered in landslide reliability analysis, the resulting failure probability is often unrealistic and overly conservative [18,12]. In fact, the spatial heterogeneity of rock and soil properties is a key factor contributing to localized instability or overall failure behavior of landslides. Building on this, incorporating the temporal evolution characteristics of landslide processes further increases the complexity of the problem. Due to long-term loading, hydrological disturbances, and other factors, the mechanical response of geotechnical bodies such as slopes or foundations exhibits significant time-dependency. Therefore, reliability analysis based solely on static assumptions often fails to meet the needs of practical engineering applications [19].

To address time-dependent reliability analysis of landslides, researchers have developed various computational methods. Traditional approaches primarily rely on Monte Carlo Simulation (MCS). Although MCS offers good general applicability and theoretical accuracy, it becomes computationally intensive when dealing with low failure probabilities, long time series, and geological bodies with significant spatial variability (such as large-scale reservoir bank landslides), making it difficult to apply efficiently in real-world engineering [15]. Under such circumstances, long-term time-dependent reliability analysis that accounts for spatial variability of rock and soil properties imposes a heavy computational burden, especially for large-scale reservoir bank landslides. Therefore, advanced approaches are needed to perform long-term reliability assessments of reservoir bank landslides while considering the spatial variability of soil properties and actual hydrological conditions.

This study aims to overcome the limitations of traditional landslide failure probability prediction methods. These conventional approaches, which typically rely on statistical regression, limit equilibrium analysis, or stability coefficient methods, often estimate failure probability based on input parameters from a single scenario. This neglects the uncertainty and variability of future environmental triggers, such as rainfall and reservoir water levels. Furthermore, these methods generally produce a single failure probability value, making it difficult to capture the uncertainty in predictions arising from fluctuations in input data. As a result, they limit a comprehensive understanding of the dynamic evolution of landslide risk.

“While random field theory captures spatial uncertainty, its computational intensity limits time-dependent applications. Deep learning offers a surrogate modeling framework that can approximate stochastic responses with significantly lower computational cost.” Against this background, this study proposes a failure probability interval prediction method driven by extreme scenarios. By defining two boundary input conditions—namely, the most unfavorable and the most favorable scenarios—and employing a deep learning model to perform parallel predictions, the method effectively quantifies the range of risk variation caused by input uncertainty. It also generates upper and lower bounds for the time-varying landslide failure probability. The predicted failure probabilities of the Baishuihe landslide range from 0.005 to 0.55, depending on the structural orientation angle and environmental conditions. Compared to traditional models, the proposed HRHN model outperforms the EA-LSTM model, with R^2^ values of 0.913 and 0.864 under the most critical and safest scenarios, respectively. These results highlight the model’s high predictive accuracy and robustness in capturing time-dependent variations in failure probability, offering enhanced risk identification capability and decision-making support, making it particularly suitable for dynamic adjustment of early warning thresholds and evolutionary monitoring of landslides under multi-source disturbances. “The proposed framework advances landslide reliability assessment by innovatively integrating rotated anisotropic random fields with deep learning–based failure probability interval prediction, enabling both spatial uncertainty and temporal evolution to be quantified simultaneously.”

## 2. Study area

### 2.1. Baishuihe landslide

**Fig 1:**
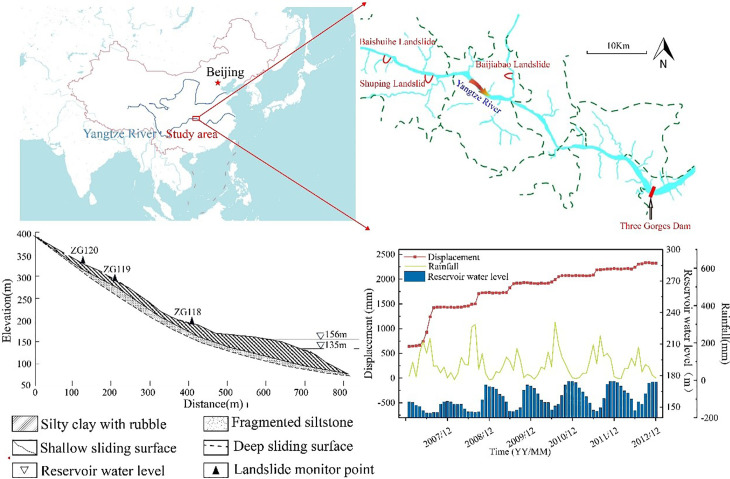
Comprehensive map of landslide location, topography, and monitoring data.

The Baishuihe landslide is located on the right bank of the Yangtze River, under the jurisdiction of Shazhenxi Town, Zigui County, Hubei Province, approximately 56 kilometers from the Three Gorges Dam. It is a typical debris-type landslide, with an average slope of 30° and an average thickness of about 30 meters. The landslide has a fan-shaped morphology, with a volume of 1.26 × 10^7^ m^3^, extending 600 meters in the north–south direction and 700 meters in the east–west direction. The main sliding direction is 20° northeast. The landslide has steep slopes at both the toe and the rear, while the central area is relatively flat. The strata have a strike of 15° and a dip angle of 36° [20]. From a flat perspective, the landslide boundary exhibits an irregular circular, armchair-like shape (Fig 1). (The data used in this study is sourced from the National Geospatial Data Cloud and Bigemap software).

Among the 11 GPS monitoring points installed within the landslide area, point ZG118 was selected as the study point for landslide displacement prediction. The monitoring curve in Fig 1 clearly shows that the displacement of the Baishuihe landslide exhibits a step-like growth pattern, which is closely related to the periodic variations in reservoir water level and rainfall. Two main movement phases are observed: from May to August each year, accompanied by heavy rainfall and low reservoir water levels, the landslide undergoes short-term rapid deformation; during other periods, with light rainfall and high reservoir water levels, the landslide remains in a long-term stable state [21].

## 3. Methods

### 3.1. LSTM with evolutionary attention mechanism (EA-LSTM)

LSTM (Long Short-Term Memory) is a type of recurrent neural network designed for time series modeling, proposed by Hochreiter and Schmidhuber in 1997. It effectively alleviates the problems of vanishing and exploding gradients commonly found in traditional RNNs when handling long sequences. By introducing gating mechanisms—namely the input gate, forget gate, and output gate—LSTM is able to retain important temporal information and has been widely applied in fields such as speech recognition, financial forecasting, and geological disaster monitoring.

However, LSTM still has two major limitations: first, it treats all input features equally, making it difficult to identify the most relevant ones for prediction; second, it struggles to capture long-range dependencies in lengthy sequences. To address these issues, researchers have explored incorporating attention mechanisms into the LSTM structure to enhance the model’s ability to focus on critical time steps and important features. One representative model is the Dual-stage Attention-based Recurrent Neural Network (DA-RNN) proposed by [22].

Building on this, [14] proposed the EA-LSTM model, which combines attention mechanisms with evolutionary algorithms for the first time. It builds an LSTM framework where attention weights can be optimized via genetic algorithms. Using Competitive Random Search (CRS), the model assigns dynamic weights to each variable and generates optimal parameter combinations, effectively capturing hidden patterns in the data and addressing the limitations of fixed attention in traditional LSTM. For more details, see [23].

### 3.2. Convolutional neural network (CNN)

Convolutional Neural Network (CNN) is one of the most representative neural network architectures in the field of deep learning, widely applied in areas such as image recognition, speech processing, and time series analysis. For example, in geotechnical engineering, CNN has been successfully used to advance reliability analysis of geotechnical infrastructure [24] and slope deformation prediction [25], among other applications.

**Fig 2:**
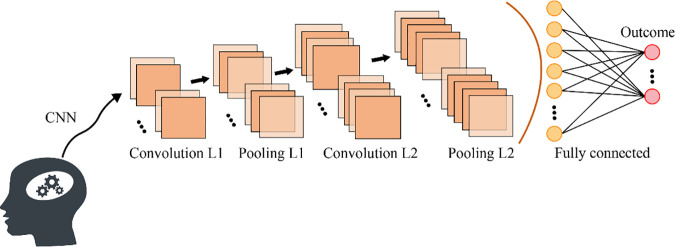
The architecture of CNN.

A typical Convolutional Neural Network (CNN) consists of six main parts: input layer, convolutional layer, activation layer, pooling layer, fully connected layer, and output layer. The input layer receives raw data such as images. Convolutional layers use filters to extract local features, activation layers (e.g., ReLU) introduce non-linearity, and pooling layers downsample to reduce dimensionality. Then, fully connected layers integrate features and map them to the target task, while the output layer generates the final prediction. This process gradually abstracts the data, improving the model’s ability to understand images and similar grid-based data (Fig 2).

### 3.3. Recurrent highway network (RHN)

Recurrent Highway Network (RHN) is an improved neural network architecture that combines the advantages of Highway Networks and Recurrent Neural Networks (RNNs). It was first proposed by [26] with the aim of enhancing the expressive power and training stability of traditional RNNs in deep structures. RHN performs particularly well in handling long sequences and complex contextual dependencies, especially in natural language processing tasks.

The core idea of RHN is to introduce multiple layers of “highway” structures within each time step, allowing the model not only to update states along the temporal dimension but also to perform deep nonlinear feature transformations at each time point. This structure employs gating mechanisms—specifically, the transform gate and carry gate—to control whether information undergoes nonlinear transformation or is directly carried over. This enables the model to maintain deep modeling capabilities while effectively mitigating the vanishing gradient problem.

RHN was developed based on Highway Networks, which were introduced by [27] to incorporate “information highways” in deep feedforward networks, thereby stabilizing training in deep architectures. Compared to traditional RNNs, LSTMs, and GRUs, RHN demonstrates higher accuracy and stronger generalization performance in tasks such as language modeling and text generation. Moreover, the design of RHN has inspired subsequent models including Depth-gated RNNs [28] and Residual RNNs (Residual RNN) [29].

**Fig 3:**
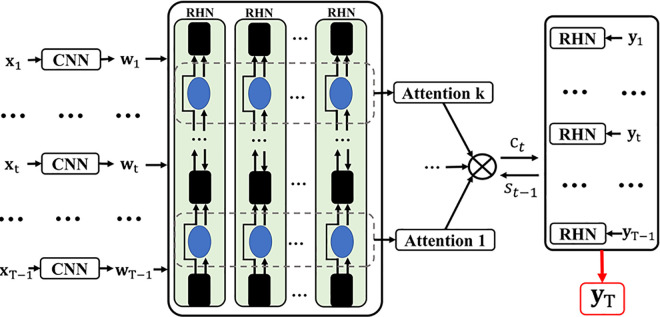
The architecture of HRHN.

The HRHN (Hierarchical Attention-based Recurrent Highway Network) model used in this study is an extension of RHN that integrates hierarchical attention mechanisms with the highway structure, as demonstrated by [30]. This approach more effectively captures multi-level dynamic features in time series, improving both predictive performance and interpretability. As shown in Fig 3, the encoder first uses a Convolutional Neural Network (CNN) to automatically learn spatial correlations among exogenous data components. Then, the RHN establishes temporal dependencies at different semantic levels, integrating time features of exogenous and target variables for effective prediction of the target value. In the decoder, the hierarchical attention mechanism selects relevant multi-level encoded semantics, while another RHN captures long-term temporal dependencies from historical observations, leveraging interactions between observations and exogenous data for the final prediction. Unlike traditional LSTM, HRHN employs hierarchical temporal representations and highway connections that prevent gradient vanishing and improve the capture of long-term dependencies, thereby improving the model’s stability and prediction accuracy in time-series learning.”

### 3.4. Reliability analysis of slope stability with spatially variable soil properties

The main idea of reliability analysis in geotechnical engineering is to quantify the safety margin of geotechnical structures using a reliability index (or an equivalent failure probability) through probabilistic methods. In this process, geotechnical parameters are modeled as random variables or random fields to characterize their uncertainty. This study considers the spatial variability of soil parameters, thus requiring random field modeling. According to random field theory, spatially variable soils can be reasonably represented by autocorrelation information (such as autocorrelation functions and correlation lengths) and certain statistical features (such as mean, coefficient of variation, and probability distribution). Common modeling techniques include the Cholesky decomposition method (or midpoint method) and the Karhunen–Loève expansion. This study adopts the midpoint method as an example and uses a squared exponential function to capture the correlation structure of spatially variable soil parameters.

Besides random field modeling, the evaluation of failure probability is also a core issue in slope stability reliability analysis. It is well known that the performance functions involved in geotechnical engineering are usually implicit and complex, making direct analytical calculation of reliability indices extremely difficult. As an alternative, various numerical methods have been proposed to assist in solving reliability indices, such as Monte Carlo Simulation (MCS). Although the computational efficiency of Monte Carlo methods is generally moderate, its flexibility and robustness make it a benchmark tool for validating other novel numerical approaches.

After generating a large number of random field samples, the study repeatedly performs slope stability analyses and repeatedly uses geotechnical engineering software to calculate multiple slope safety factors. Finally, the failure probability $P_{f}$ can be calculated as:


$$P_{f}=\frac{1}{N}\sum_{i=1}^{N}I[FS({\overset{̑}{X}}_{i}<1)]$$ (1)

Where N is the number of iterations, and the FS value corresponds to the i-th set of random variable samples; I[·] is the indicator function used to determine whether slope failure occurs—if $FS({\overset{̑}{X}}_{i})<1$, then I[·] =1, otherwise, I[·] =0.

### 3.5. Reliability analysis based on deep learning

The stability of reservoir slopes in the Three Gorges Reservoir area often varies with changes in external conditions, such as seasonal rainfall and periodic fluctuations in reservoir water levels. Therefore, to better capture temporal characteristics, time-dependent reliability analysis of reservoir slope stability is necessary. Although the methods mentioned above can be directly applied to perform time-dependent reliability analysis by discretizing the problem at discrete time points into a series of time-independent reliability analyses, the computational workload can be very large and time-consuming, especially when considering spatially variable soil. In this context, deep learning offers a reverse approach to learn the temporal information behind time series data and further predict target values over a future horizon in a data-driven manner. For a one-step ahead prediction model, the predicted target value ${\overset{̑}{y}}_{t+1}$ at time t + 1is calculated by the following equation:


$${\overset{̑}{y}}_{t+1}=f(y_{t-k:t},x_{t-k:t},s)$$ (2)

Here, $y_{t-k:t}=\left(y_{t-k},y_{t-k+1},\cdots ,y_{t}\right)$ represents the target values over a predefined look-back window k, which determines how much historical information is considered; $x_{t-k:t}=\left(x_{t-k},x_{t-k+1},\cdots ,x\right)$ denotes the inputs within the look-back window k; and s represents relevant static data. f(·) is the deep learning prediction model. It can be seen that data preparation for the prediction model forms the foundation of time series forecasting.

The preparation of data for model training and testing is a prerequisite for building deep learning (DL) models. Unlike common time series prediction problems in geotechnical engineering—such as landslide displacement forecasting and surface subsidence monitoring—where target data are measurable, the reliability index in geotechnical engineering is an unmeasurable target that requires geotechnical reliability analysis. Therefore, data preparation is a major focus of this study.

To obtain the training and testing data needed for the deep learning model, this study develops an automated workflow using Python to integrate finite element modeling and random parameter assignment based on GEOSTUDIO software and random field theory. First, a slope finite element model is constructed, and the coordinates of the mesh centroids are extracted to generate spatially correlated random fields of soil parameters. Then, multiple sets of parameter samples are generated using Latin Hypercube Sampling combined with probability transformation, and these are assigned in batches to the model. Using the Monte Carlo Simulation (MCS) method, multiple simulations are performed at each time point to extract the corresponding safety factors, from which the failure probability at that moment is statistically obtained. The final result is a time series of failure probabilities that can be used for deep learning modeling.

The DL-based time-dependent reliability analysis method not only enables geotechnical engineers to efficiently capture the stability status of reservoir slopes affected by seasonal rainfall and periodic reservoir water level fluctuations but also provides important reference value for decision-makers in designing reasonable mitigation measures for landslide disaster management. The detailed implementation process is arranged in subsequent sections.

## 4. Implementation process and prediction results

### 4.1. Implementation procedure

**Fig 4:**
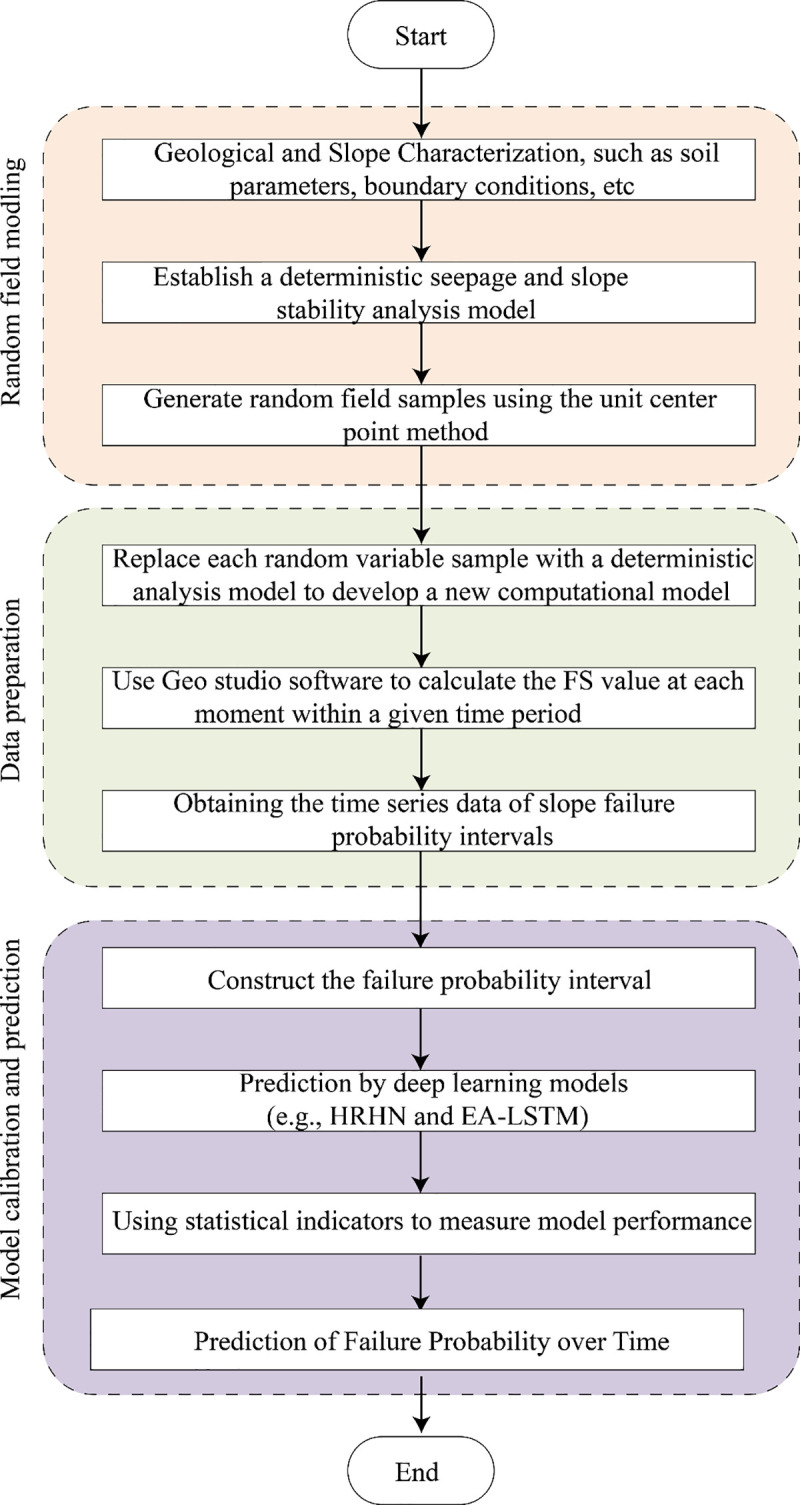
Flow chart of the time-dependent reliability analysis using deep learning (DL).

The method proposed in this study consists of three key steps: random field modeling, preparation of training and testing data, and time series-based deep learning prediction (Fig 4). First, a random field model is constructed based on the statistical characteristics of soil parameters (such as probability distributions) and boundary conditions (such as rainfall intensity and reservoir water levels). Then, using the element centroid method, a large number of random field samples satisfying spatial correlation are generated. For each sample set, multiple deterministic finite element analyses are conducted, repeatedly invoking geotechnical software to calculate the corresponding safety factors (FS).

Taking GeoStudio as an example, Python scripts are used to batch-operate its SEEP/W and SLOPE/W modules, enabling automated processing of N sets of input files. The simulation results are stored in corresponding folders. From each folder, safety factors are extracted and compiled into a series corresponding to the N random field samples, ultimately forming a time series of failure probabilities.

Based on this time series data, two deep learning models are used to predict slope failure probability: The Hierarchical Attention-based Recurrent Highway Network (HRHN) and the improved Evolutionary Attention-based LSTM (EA-LSTM). Experimental results show that the HRHN model performs exceptionally well in capturing complex temporal dependencies and multi-scale features, significantly outperforming the traditional EA-LSTM model in both prediction accuracy and generalization capability.

Finally, by combining the HRHN model predictions under the most unfavorable and most favorable scenarios, an interval trend of failure probability for the Baishuihe landslide is constructed. This enables a deeper understanding of the dynamic evolution of landslide risk over time. Compared to traditional prediction methods that provide only a single failure probability, the interval-based approach reflects the uncertainty in the prediction results, offering a more comprehensive understanding of risk and enhancing the scientific basis and reliability of landslide disaster early warning and prevention strategies.

To demonstrate its effectiveness, the proposed method is applied to the Baishuihe landslide case.

### 4.2. Effect analysis of random field parameters

**Fig 5:**
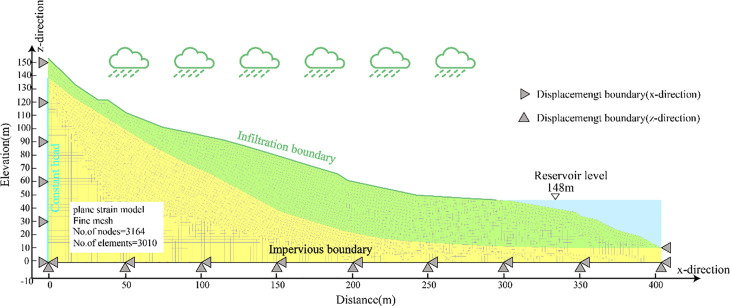
Seepage and stability model of the baishuihe landslide.

A two-dimensional finite element seepage and stability analysis model of the Baishuihe landslide cross-section, as presented in the text, was constructed using the GEOSTUDIO software (Fig 5). The SEEP/W module was used to establish the finite element seepage model shown in the figure, in which the landslide body area was divided into a total of 1,183 finite element mesh units. The area above the groundwater level within the landslide body was set as a rainfall boundary, the rear edge was defined with a constant head boundary (head value of 100 m), and the bottom boundary was specified as impermeable.

In engineering practice, determining the coefficient of variation (COV), probability distribution, autocorrelation function, and scale of fluctuation (SOF) for random field modeling is not straightforward, as available measurements are often sparse and limited. In this example, the statistical information was collected from previous studies. [31,32], For simplicity, among several parameter models of the soil-water characteristic curve (SWCC), the van Genuchten SWCC is used to characterize the hydraulic behavior of unsaturated soils. Tables 1 and 2 summarize the parameters used in this example.

**Table 1:** The values of physical and mechanical parameters of rock and soil mass.

Parameters	Landslide mass	Bed rock
cohesion (kPa)	27	100
Permeability coefficient (m/s)	$\text{5}\times {\text{10}}^{-\text{6}}$	$\text{5}\times {\text{10}}^{-\text{8}}$
internal friction angle (°)	18	32
Poisson’s ratio	0.31	0.31
Elastic modulus (MPa)	35.6	100
unit weight (kN/$m^{\text{3}}$)	21	26

**Table 2:** Van Genuchten model fitting parameters of the landslide mass.

Parameters	a(kPa)	*n*	Sat.WC	Rse.WC
Value	40	1.45	0.43	0.02

Specifically, these include: (1) Basic physical and mechanical parameters: mean values of unit weight (γ), cohesion (c), internal friction angle (φ) and saturated hydraulic conductivity ($k_{s}$) of the geotechnical material; (2) Mean values representing key fitting parameters of the van Genuchten model: fitting parameter (a), shape parameter (n), saturated water content $\theta_{s}$ (Sat.WC), and residual water content $\theta_{r}$ (Rse.WC).

For the random field parameters such as the coefficient of variation, cross-correlation coefficients, scale of fluctuation, and directional structure of cohesion, internal friction angle, and permeability coefficient, previous studies have shown that [16,33,34] the coefficients of variation (COV) of the strength parameters (cohesion c and internal friction angle φ) and permeability coefficient $k_{s}$ as well as the correlation coefficient $\rho_{c,\varphi }$ between c and φ, all influence the probability distributions of these parameters, controlling their variability and directly affecting their positive correlation with the slope failure probability $P_{f}$ [35]. The scales of fluctuation ($\delta_{max}$ and $\delta_{\text{min}}$) and the horizontal structural orientation angle $\theta_{\delta_{min}}^{GA}$ affect the frequency of alternating zones of high-strength and weak soil within the slope, thereby causing variations in failure probability. “In reservoir areas, the geological bedding or structural planes often deviate from the horizontal, resulting in rotated anisotropic variability that governs the mechanical behavior of colluvial deposits.” Among these, the non-horizontal structural orientation angle $\theta_{\delta_{max}}^{GA}$, is the most sensitive factor influencing the slope failure probability. This orientation angle determines the spatial distribution trend of weak zones along the main sliding direction within the soil mass and is a key controlling factor affecting the formation of potential failure paths and the scale of sliding. In contrast, although the variability and correlation of strength parameters significantly impact parameter dispersion and local weakening, they mainly influence the formation of local failure modes rather than the overall landslide failure mechanism. Their effect on the global failure process is less direct and pronounced compared to that of the structural orientation angle. Therefore, when modeling failure probability for slopes with non-transversal anisotropy, special attention must be paid to the selection and simulation of the structural orientation angle to more accurately assess potential risks.

**Fig 6:**
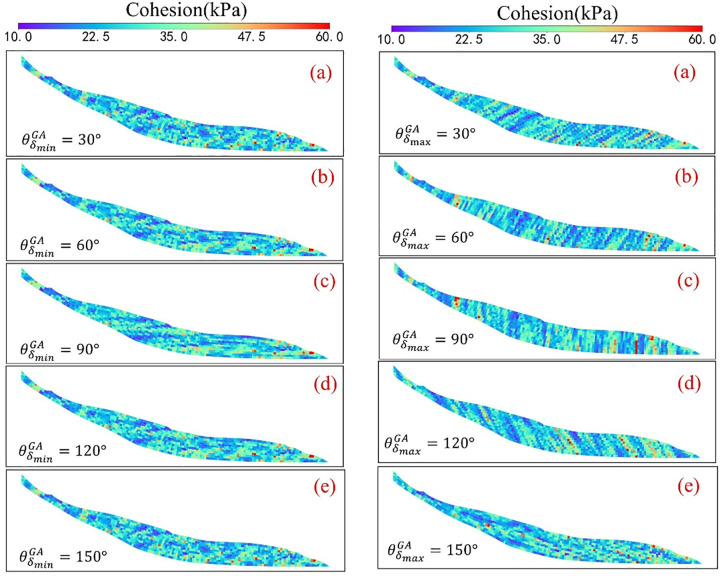
Influence of Horizontal and Non-Horizontal principal directions in the anisotropic random field of friction angle on landslide failure probability.

Next, the influence of the structural orientation angle on slope reliability was systematically studied under conditions of general anisotropic spatial variability, focusing separately on horizontal and non-horizontal structural scenarios. The study explored the controlling mechanisms of the structural angle on landslide failure probability within the range of 30° to 150° (Fig 6). In the horizontal structure scenario, by varying the horizontal structural orientation angle $\theta_{\delta_{min}}^{GA}$, it was found that its effect primarily controls the spatial distribution frequency of strength layers. When $\theta_{\delta_{min}}^{GA}=\text{9}\text{0}{}^{\circ }$ (representing transverse anisotropy), the failure probability $P_{f}$ reaches its maximum and exhibits clear symmetry with respect to changes in the orientation angle. This indicates that under transverse anisotropy, weak zones within the soil are more likely to form continuous distributions, increasing the risk of slope failure.

**Fig 7:**
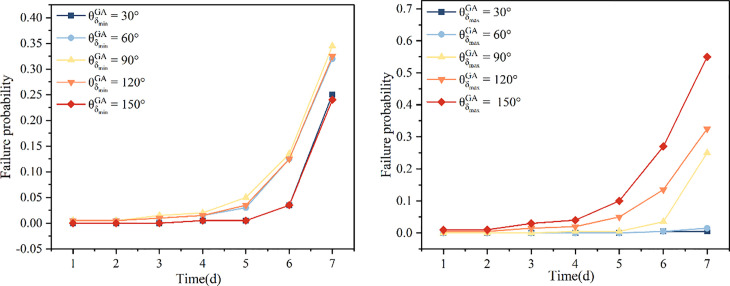
Effect of structural direction angle on slope reliability (Horizontal vs. Non-Horizontal).

In the non-horizontal structure scenario, variations in the non-horizontal structural orientation angle $\theta_{\delta_{max}}^{GA}$ not only affect the spatial distribution direction of strength layers but also directly determine their coupling relationship with the slope surface. The study shows that when $\theta_{\delta_{max}}^{GA}=\text{1}\text{5}\text{0}{}^{\circ }$, meaning the orientation is nearly parallel to the slope surface, a dipping structure forms that significantly enhances the connectivity of weak zones along the slope, causing the failure probability to peak at $P_{f}=\text{0}.\text{5}\text{5}$. Conversely, when $\theta_{\delta_{max}}^{GA}=\text{3}\text{0}{}^{\circ }$, an anti-dip structure forms that restricts the sliding path, resulting in the highest slope reliability $\left(P_{f}=\text{0}.\text{0}\text{0}\text{5}\right)$. Compared to the peak failure probability in the horizontal structure scenario ${(P}_{f}=\text{0}.\text{3}\text{4}\text{5})$, the most adverse case caused by the non-horizontal structure nearly doubles this value, indicating that under general anisotropic conditions, the non-horizontal structural orientation angle has the most significant impact on landslide failure probability (Fig 7). Especially when soil layers exhibit a dipping distribution trend, reliability assessment cannot be simplified to the traditional transverse anisotropy assumption; otherwise, the potential sliding risk will be severely underestimated.

The above analysis indicates that when considering general anisotropic spatial variability, variations in the structural orientation angle can cause significant fluctuations in failure probability. This effect is especially pronounced in non-horizontal structural scenarios, where the geometric relationship between the structural angle and the geological interfaces or bedding directions of the slope has a decisive impact on landslide stability. In practical engineering, landslide masses are often influenced jointly by strata orientation, the trend of weak interlayers, and geostress disturbances, making the structural orientation angle uncertain or difficult to determine precisely. Consequently, relying on failure probability predictions based on a single scenario (such as a fixed structural angle, rainfall, or water level condition) in slope reliability assessments cannot comprehensively capture the full range of risk, often resulting in overly conservative or non-conservative judgments.

Therefore, to more realistically reflect the multiple uncertain scenarios that slope systems may face under geological structures and environmental disturbances, this study further proposes a failure probability interval prediction method based on extreme condition settings. Other random field parameters (such as coefficients of variation, correlation coefficients, etc.) are fixed at the standard values listed in Table 3, while $\theta_{\delta_{max}}^{GA}$ is treated as a variable. Two extreme scenarios are constructed: (1) $\theta_{\delta_{max}}^{GA}={\text{1}\text{5}\text{0}}^{\circ }$, representing the “most dangerous case” that induces the maximum failure probability; (2) $\theta_{\delta_{max}}^{GA}={\text{3}\text{0}}^{\circ }$, representing the “safest case” that results in the minimum failure probability. This setup allows for a systematic analysis of the evolution of time-dependent reliability of the slope under these two scenarios.

**Table 3:** Standard values of random field parameters.

Model parameters	Correlation coefficient	Coefficient of variation	Non-Horizontal orientation angle(°)	Maximum fluctuation length(m)	Minimum fluctuation length(m)
Strength parameter	0	0.3	30 ~ 150	40	4
Permeability coefficient	–	0.3	30 ~ 150	40	4

To obtain the time-dependent reliability, this study also takes the Baishuihe landslide in the Three Gorges Reservoir area as the research subject and employs the HRHN model to predict the temporal evolution of the landslide failure probability. The model input parameters are listed in the Table 4. The time span is from December 2007 to December 2012, covering a total of 5 years (60 months), with monthly data recorded. For each month, 200 random field simulations were conducted, resulting in a total of 12000 model runs. By modeling these two types of dynamic inputs with high spatiotemporal resolution, the HRHN model can identify the nonlinear response patterns of the landslide system to environmental changes, thereby enabling more accurate predictions of failure probability.

**Table 4:** Input variables for failure probability interval.

Input Variable	Fixed/Varying	Engineering Significance
Non-horizontal structure angle	Varying	Controls principal direction of spatial variability
Internal friction angle	Fixed	Key parameter affecting shear strength
Cohesion	Fixed	Controls shear resistance and stability
Unit weight	Fixed	Influences gravitational force and pore pressure
Spatial correlation length	Fixed	Controls scale of spatial variability
Coefficient of variation	Fixed	Determines the degree of parameter randomness
Rainfall intensity	Varying	Increases driving force and softens material
Reservoir drawdown rate	Varying	Reduces hydrostatic pressure, may trigger instability

### 4.3. Prediction performance evaluation metrics

Two metrics are used to evaluate prediction performance, including the Root Mean Square Error (RMSE) and the Coefficient of Determination ($R^{\text{2}}$):


$$RMSE=\sqrt{\frac{\text{1}}{T}\sum_{t=1}^{T}{\left(y(t)-\hat{y}(t)\right)}^{2}}$$ (3)

$$R^{2}=1-\frac{\sum_{t=1}^{T}{\left(y(t)-\hat{y}(t)\right)}^{2}}{\sum_{t=1}^{T}{\left(y(t)-\bar{y}(t)\right)}^{2}}$$ (4)

Here, *T* is the number of samples in the test dataset; y(t) and $\bar{y}(t)$ represent the observed displacement and its mean value, respectively; $\hat{y}(t)$ is the predicted value. RMSE and $R^{\text{2}}$ are used to evaluate the prediction accuracy of landslide displacement points. The smaller the RMSE, and the larger the $R^{\text{2}}$ the better the prediction performance.

### 4.4. Time-dependent failure probability analysis

**Fig 8:**
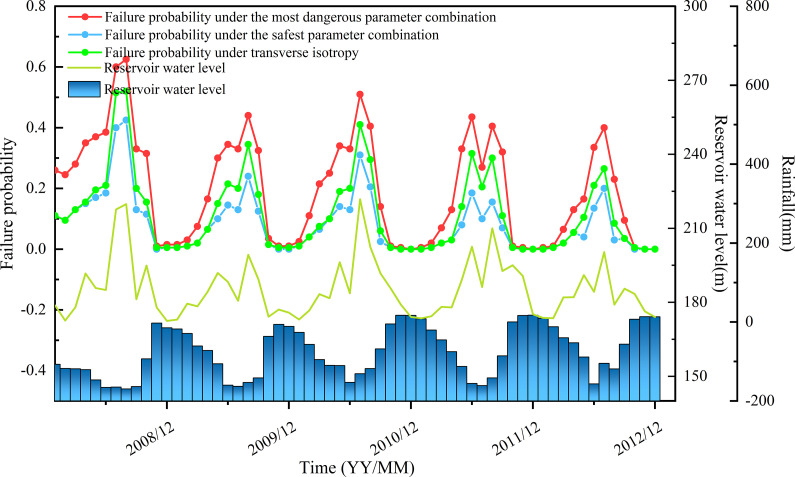
Time-dependent failure probability of the baishuihe landslide.

Fig 8 illustrates the temporal variation of the failure probability $P_{f}$ of the Baishuihe landslide from 2008 to 2012. This variation is represented as an interval composed of the maximum and minimum failure probability scenarios obtained from numerical simulations under general anisotropy, and is compared with a single-curve evolution derived from transversely isotropic assumptions. According to [36], the deformation of the Baishuihe landslide is positively correlated with fluctuations in the reservoir water level; specifically, the greater the rate of water level decline, the higher the landslide deformation rate. When the reservoir water level rises, the slope experiences an increased hydrostatic load, along with enhanced hydrodynamic pressure acting inward on the slope, which improves the soil’s shear resistance and thus reduces the failure probability $P_{f}$. Conversely, when the reservoir water level decreases, the slope load is reduced, and seepage induces outward drag forces that weaken the soil skeleton stability, increasing the risk of slope failure [37], thereby causing $P_{f}$ to rise. Notably, from May to August each year, the reservoir water level drops rapidly, coinciding with the rainy flood season. Rainfall infiltration on one hand increases the soil’s unit weight, intensifying the driving force due to gravity; on the other hand, it softens the sliding mass materials and reduces their shear strength, further exacerbating the increase in failure probability [37]. The time-dependent features of $P_{f}$ shown in Fig 8 strongly align with the above analysis: months with declining water levels combined with rainfall infiltration (e.g., May to August) exhibit significant increases in $P_{f}$; whereas during periods of rising or stable water levels, $P_{f}$ tends to decrease. This trend is consistent with findings from other researchers [38,39,12].

Furthermore, a close examination of Fig 8 reveals a strong coupling relationship between the failure probability interval under general anisotropy and the single-curve prediction based on transverse isotropy. Throughout the observation period, the transverse isotropy curve consistently lies within the interval and closely follows its temporal trend. During high-risk periods such as the flood season, all three curves—the upper bound, lower bound, and the transversely isotropic prediction—exhibit a simultaneous upward trend, indicating that the failure probability interval effectively captures the overall temporal evolution trend of landslide instability. However, during low-risk periods, the transversely isotropic prediction often lies closer to the lower bound of the interval, reflecting a conservative bias and a limited capacity to capture extreme risk scenarios. This underscores the added value of interval-based prediction, which more comprehensively represents the range of potential outcomes under parameter and geological uncertainty.

Compared to a single deterministic prediction, the use of a failure probability interval offers multiple advantages. First, the interval provides not only upper and lower bounds for the prediction but also reveals the range of variability caused by parameter uncertainty and geological heterogeneity, thereby improving the robustness of risk assessments. Second, the interval captures transitions between high-risk and low-risk stages more clearly; when the upper bound approaches the critical threshold, it can support timely early warning and preventive actions. When the interval narrows and both bounds indicate low risk, it provides a scientific basis for optimizing resource allocation and control measures.

Additionally, the interval-based approach demonstrates superior sensitivity to complex environmental factors compared to traditional single-curve predictions under transverse isotropy. It avoids potential underestimation or misjudgment of risk due to oversimplified assumptions.

### 4.5. Comparison of methods based on deep learning (DL)

**Fig 9:**
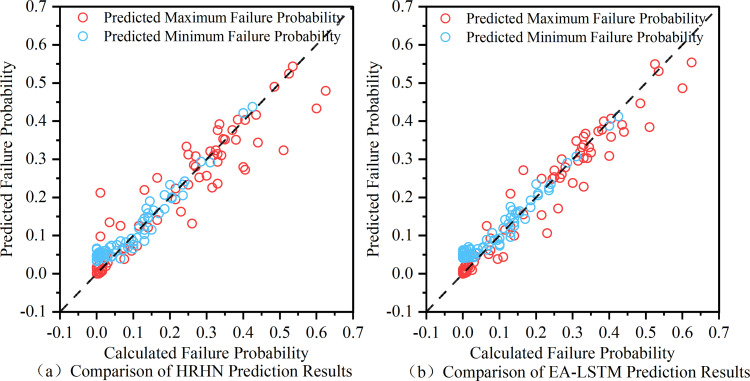
Comparison of predictions from two models with actual values.

Fig 9 compares the failure probability $P_{f}$ of the Baishuihe landslide calculated by the Stochastic Finite Element Method (SFEM) with the failure probabilities predicted by the HRHN and EA-LSTM models. The results show that the predictions from both models agree well with the SFEM calculations. Most data points cluster around the 1:1 reference line, indicating that both HRHN and EA-LSTM can predict the failure probability $P_{f}$ of the Baishuihe landslide with high accuracy. This consistency validates the effectiveness of the models in capturing the time-dependent reliability of the landslide, providing reliable support for their application under complex hydrogeological conditions.

To quantitatively assess the predictive performance of the two deep learning (DL) models, Table 5 presents the evaluation results of the HRHN and EA-LSTM models for time-dependent failure probability prediction of the Baishuihe landslide under the most critical scenario ($\theta_{\delta_{max}}^{GA}={\text{1}\text{5}\text{0}}^{\circ }$) and the safest scenario ($\theta_{\delta_{max}}^{GA}={\text{3}\text{0}}^{\circ }$). The results indicate that the HRHN model outperforms the EA-LSTM model under both scenarios. Specifically, under the most critical scenario, the HRHN model achieved an $R^{\text{2}}$ of 0.913, and an RMSE of 0.057, whereas the EA-LSTM model yielded an $R^{\text{2}}$ of 0.870 and an RMSE of 0.069;Under the safest scenario, the HRHN model obtained an $R^{\text{2}}$ of 0.864 and an RMSE of 0.046, compared to the EA-LSTM’s $R^{\text{2}}$ of 0.851 and RMSE of 0.050. The HRHN model’s $R^{\text{2}}$ values are closer to 1 and its RMSE values are lower, indicating a higher degree of fit between predicted and actual values and smaller prediction errors, reflecting superior overall performance. Therefore, for the time-dependent failure probability prediction of the Baishuihe landslide, the HRHN model performs better than the EA-LSTM model. This implies that the temporal features of the time series data are reasonably and accurately captured by the HRHN model. Consequently, it can be concluded that the HRHN model demonstrates superior capability in predicting the temporal variation of failure probability for the Baishuihe landslide case compared to the EA-LSTM model.

**Table 5:** Model evaluation metrics.

Working condition	Models	*R* ^2^	*RMSE*
Most critical scenario	HRHN	0.913	0.057
	EA-LSTM	0.870	0.069
Safest scenario	HRHN	0.864	0.046
	EA-LSTM	0.851	0.050

**Fig 10:**
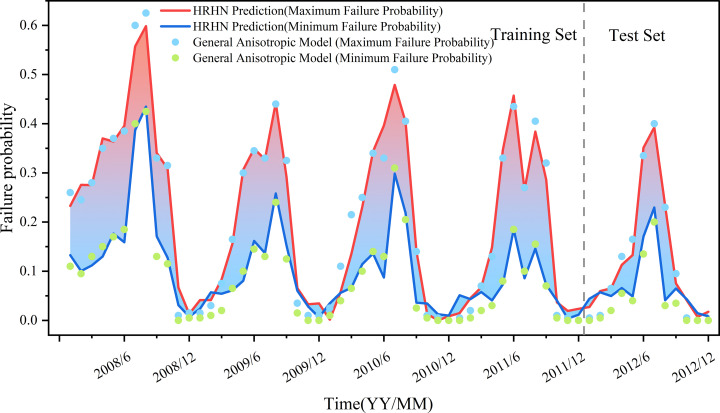
Evolution of failure probability predicted by the HRHN model.

Fig 10 presents the interval evolution trend of the time-dependent failure probability $P_{f}$ of the Baishuihe landslide predicted by the HRHN model. This interval compares the interval evolution predicted by the HRHN model with the failure probability range constructed from the most dangerous scenario ($\theta_{\delta_{max}}^{GA}={\text{1}\text{5}\text{0}}^{\circ }$) and the minimum failure probability scenario ($\theta_{\delta_{max}}^{GA}={\text{3}\text{0}}^{\circ }$) under general anisotropy. The interval evolution of failure probability effectively captures the significant influence of rainfall and reservoir drawdown on landslide instability. The interval evolution of failure probability effectively captures the significant influence of rainfall and reservoir drawdown on landslide instability.

The results shown in the figure indicate that the HRHN model effectively captures the periodic fluctuation characteristics of failure probability, with the predicted values generally aligning well with the actual trends. This is especially evident during the high failure probability periods in the flood season (May to August), reflecting the significant impact of rainfall and reservoir water level decline on landslide instability. The model reasonably characterizes the dynamic variation trend of failure probability, particularly during the trough and rising phases, where deviations between predicted and actual values are minimal, demonstrating strong generalization capability.

However, the figure also reveals some periods where the minimum failure probability exceeds the maximum failure probability, notably during low failure probability intervals (e.g., December 2009, December 2010), when failure probabilities are generally below 0.1. This anomaly primarily stems from the limitations of the HRHN model as a deep learning approach in predicting low-probability scenarios, possibly due to insufficient training data or limited generalization ability for low-risk conditions. Nonetheless, since the landslide risk during these periods is inherently very low, this anomaly has minimal impact on the overall risk assessment and practical decision-making for the Baishuihe landslide Therefore, despite some instability in prediction within low-probability regions, the HRHN model’s results still effectively support early warning and management during high-risk periods.

Overall analysis demonstrates that the HRHN model can more accurately depict the time-varying characteristics of failure probability for the Baishuihe landslide, exhibiting high predictive accuracy and generalization. The interval evolution trend of time-dependent failure probability $P_{f}$ established by this model also provides a reliable tool for dynamic risk assessment of landslides in the Three Gorges Reservoir area. This outcome not only advances the theoretical study of time-dependent landslide reliability but also offers important references for disaster prevention and mitigation design. “Although this study focuses on the Baishuihe case, the framework can be generalized to other reservoir slopes with similar hydrological boundary conditions.”

## 5. Summary and conclusions

This study combines the Stochastic Finite Element Method (SFEM) with GEOSTUDIO software and anisotropic random fields to assess the impact of spatial variability on slope reliability, validated through the Baishuihe landslide case study. A systematic analysis of the time-dependent reliability of the landslide was conducted. First, by introducing spatial variability characterization and incorporating the maximum fluctuation scale orientation angle $\theta_{\delta_{max}}^{GA}$, two extreme scenarios were constructed: the “most critical scenario” ($\theta_{\delta_{max}}^{GA}={\text{1}\text{5}\text{0}}^{\circ }$) and the “safest scenario” ($\;\theta_{\delta_{max}}^{GA}={\text{3}\text{0}}^{\circ }$) These scenarios reveal the dynamic evolution law of failure probability $P_{f}$ in response to reservoir water level decline and rainfall infiltration, showing a pronounced increasing trend particularly during the flood season (May to August).

Second, a deep learning model, HRHN, was proposed for predicting the time-dependent reliability of slopes and was compared with the EA-LSTM model, verifying the superior performance of HRHN in predicting time-dependent failure probability. The HRHN model effectively captures periodic fluctuations and abrupt changes in the time series, achieving higher predictive accuracy in both training and testing datasets (with $R^{\text{2}}$ values of 0.913 and 0.864 and RMSE values of 0.057 and 0.046, respectively).

The results indicate that the HRHN model exhibits strong applicability and stability in dynamic risk assessment of landslides under complex hydrogeological conditions. The interval evolution trend of time-dependent failure probability $P_{f}$ established for the Baishuihe landslide provides theoretical support and practical reference for time-dependent reliability analysis and engineering disaster prevention in the Three Gorges Reservoir area. This study reveals the time-dependent characteristics of slope stability and demonstrates the significant advantages of using deep learning to predict slope time-dependent reliability, particularly in capturing risk dynamics and supporting early warning during high-risk periods. But this study assumes stationary spatial variability and uniform boundary conditions, which may limit applicability under rapidly changing rainfall infiltration or heterogeneous stratigraphy. Future research should integrate field monitoring data and 3D random field simulation for adaptive model updating.

## Supporting information

S1 File The data used in this study. (XLSX)
